# Friend or Foe? Locoregional Therapies and Immunotherapies in the Current Hepatocellular Treatment Landscape

**DOI:** 10.3390/ijms241411434

**Published:** 2023-07-14

**Authors:** Shamar Young, Jack Hannallah, Dan Goldberg, Tina Sanghvi, Junaid Arshad, Aaron Scott, Gregory Woodhead

**Affiliations:** 1 Department of Medical Imaging, Division of Interventional Radiology, University of Arizona, 1501 N Campbell Ave, Tucson, AZ 85724, USA; 2 Department of Radiology, Southern Arizona VA, Tucson, AZ 85723, USA; 3 Department of Medicine, Division of Hematology and Oncology, University of Arizona, Tucson, AZ 85724, USA

**Keywords:** transarterial radioembolization, transarterial chemoembolization, ablation, checkpoint inhibitors, immunotherapies, hepatocellular carcinoma

## Abstract

Over the last several decades, a number of new treatment options for patients with hepatocellular carcinoma (HCC) have been developed. While treatment decisions for some patients remain clear cut, a large numbers of patients have multiple treatment options, and it can be hard for multidisciplinary teams to come to unanimous decisions on which treatment strategy or sequence of treatments is best. This article reviews the available data with regard to two treatment strategies, immunotherapies and locoregional therapies, with a focus on the potential of locoregional therapies to be combined with checkpoint inhibitors to improve outcomes in patients with locally advanced HCC. In this review, the available data on the immunomodulatory effects of locoregional therapies is discussed along with available clinical data on outcomes when the two strategies are combined.

## 1. Introduction

Hepatocellular carcinoma (HCC) is the most common form of primary liver cancer as well as the fifth most frequent malignancy and the third leading cause of cancer death worldwide [1]. In 2020, there were an estimated 906,000 new cases of primary liver cancer and 83,000 fatalities worldwide of which 75 to 85% were secondary to HCC [2]. The treatment of HCC has always been a challenge given the frequent presence of cirrhosis, which is a competing factor for survival. In fact, it was not until the 21st century that viable systemic therapies were elucidated for this challenging disease in the form of tyrosine kinase inhibitors [3,4]. Even after the efficacy of tyrosine kinase inhibitors were established, their benefit was limited, with improved overall survival (OS) over placebo of 2 to 3 months [3,4]. However, over the last decade, immunotherapies have been shown to benefit HCC patients and have become a first-line systemic therapy [5,6,7].

Given the modest benefit of systemic therapies through much of the last 50 years in combination with the fact that only 5 to 10% of patients with HCC are considered to be surgical candidates, the development of locoregional therapies became a major area of focus [8]. Locoregional therapies (including transarterial chemoembolization (TACE), ablation, and transarterial radioembolization (TARE)) have been applied in a number of settings, including curative intent [9,10], palliative, but life-prolonging, treatment [8,9,10,11,12,13], and to downstage or bridge with transplant or resection [14,15,16]. This has led to an exciting time where multidisciplinary teams have a variety of treatment options to offer patients, but this also leads to questions around how best to optimize and sequence treatment modalities under complex patient circumstances.

Herein, the authors review the available evidence for various HCC patient populations with an emphasis on the possibility of synergy between locoregional therapies and systemic therapy in a subset of HCC patients.

## 2. Categorizing HCC Patients

Categorizing HCC patients has always been problematic given the frequent presence of cirrhosis along with HCC. This leads to competing risk factors for death and can make treatment decisions difficult as the need to preserve normal liver function while at the same time eliminating the cancer is a frequent paradigm treating physicians face. A number of different algorithms have been suggested; however, the most widely adapted algorithm perhaps is the Barcelona Clinic Liver Cancer (BCLC) system, which classifies patients into five categories. The authors acknowledge the importance of underlying liver function and patient performance status; however, if these are combined with disease burden, the number of subcategories of HCC patients can become exhaustive and tedious, not to mention controversial. Therefore, for the purposes of this review, the authors have chosen to focus on HCC disease burden.

## 3. Locoregional Therapies Patient Population

The vast majority of locoregional HCC trials have been conducted in patients who do not have extra-hepatic disease, many focusing on those with a relatively low level of disease [9,10,11,12,13,14,15,16,17,18,19,20,21,22]. Several randomized controlled trials have shown the ability of thermal ablation to achieve equivalent oncologic outcomes while reducing the morbidity in small HCCs (≤3 cm) when being compared to surgical resection [9,10]. Furthermore, several recent studies have evaluated the ability of TARE to induce complete pathologic necrosis in studies, which included patients with single HCCs up to 8 cm [17,18,19,20]. The ability to have curative intent in the setting of a single HCC of up to 8 cm in combination with a relatively low complication profile leaves this group of patients still primarily receiving locoregional therapy in most centers.

## 4. Systemic Therapies’ Patient Population

Conversely, the vast majority of trials evaluating systemic therapy have been carried out in populations where a large percentage of patients have extra-hepatic disease [3,4,5,6,7,23,24,25,26,27]. For instance, the IMbrave 150 trial, which established atezolizumab and bevacizumab as first-line systemic therapy for HCC patients, included a population where the majority had extra-hepatic disease (60.9%) [5]. Therefore, in patients with confirmed extra-hepatic disease, systemic therapy has become the bedrock of treatment strategies. While initially these strategies largely included tyrosine kinase inhibitors, current first-line therapy is typically centered around immunotherapies with tyrosine kinase inhibitors having a place in second-line therapy at times [5].

## 5. Patient Population Overlap

The group of patients that are most typically considered for both locoregional and systemic therapy are those with locally advanced HCC. The term locally advanced HCC has been applied broadly; however, most would consider those outside Milan criteria (>3 HCCs < 3 cm or 1 HCC > 5 cm) to be locally advanced. Although it should be noted that in the majority of published studies of locally advanced HCC, average tumor size was much larger than 5 cm (often ≥ 10 cm) [5,13]. A notable subset of this cohort are those patients with macrovascular invasion, which again tends to represent a significant portion of those enrolled in “locally advanced” trials [5,13]. While highlights of the data for patients that have been treated by locoregional therapy or systemic therapy will be reviewed here for this population, a large emphasis will be placed on the rationale and early data to support the use of a combination of locoregional therapies and immunologically active therapies in these patients.

## 6. Rationale for a Combined Approach

While some have couched the modern treatment landscape as systemic, in particular immunotherapy, versus locoregional therapies, this approach is not necessarily the best way forward. When simply looking at immunotherapies, we know that a significant proportion of HCC patients will not respond to therapy. For example, some studies have shown a 15 to 20% response rate to immune checkpoint inhibitors such as nivolumab and pembrolizumab, with 1 to 5% experiencing a complete remission [6]. Similarly, in a phase Ib study, 20% of patients on pembrolizumab maintained a progression-free survival (PFS) of greater than a year [24]. Therefore, it is unsurprising that when larger phase III trials were performed, the median PFSs tended to be less than desired. For instance, the median PFS in IMbrave 150 was 6.8 months (95% CI: 5.7–8.3 months), and the estimated 12-month survival was 67.2% (95% CI: 61.3–73.1%) [5]. While these numbers compared favorably to the sorafenib control group, they also emphasize the need for further innovation in this cohort of difficult-to-treat patients. Furthermore, they seem to be fairly representative of the overall data available for immunotherapies. In another example, the CheckMate 040 trial demonstrated a 49% disease control rate (DCR), a 31% overall response rate (ORR), and a 17-month median duration of response [23]. This has led many to wonder if combining these systemic therapies with other treatment strategies may provide greater depth and length of response.

When looking at locoregional therapies, TARE has emerged as a treatment favorite among the three commonly used modalities (thermal ablation, TACE, and TARE) in this cohort. While external beam radiation is also considered a locoregional therapy, the significant toxicity profile (≥10% of patients developing grade 3 or greater toxicities in the majority of studies) has typically relegated its use to research settings or clinical scenarios that fall out of the norm [28,29]. TARE has emerged as front-line therapy in most institutions based on recent trials demonstrating superior PFS and OS when compared to TACE, formerly the primary locoregional therapy to be used in this setting [30,31]. However, it should be noted that there are regional differences, and TARE is not commercially available in all parts of the world, leaving TACE as a mainstay of locoregional therapy in these geographic areas. Ablation, as a standalone treatment, is typically not applied to these larger lesions given the inability to completely ablate the lesion and fears of upregulation of hypoxia inducible factor 1 alpha (HIf-1alpha) and subsequent transformation of the biologic aggressiveness if incomplete ablation is achieved. This phenomenon has been reported with thermal ablation and specifically in the case of radiofrequency ablation (RFA) [32,33,34]. However, with new forms of ablation being developed and other forms immunomodulatory benefits being better understood, this may not be true in the future.

When looking at the use of TARE in the setting of locally advanced disease, a recent randomized controlled trial comparing two dosing techniques showed a median OS of 26.6 months in a cohort with a mean tumor size of 10.9 ± 2.57 cm and in which 75% had macroscopic vein invasion [13]. In the case of TACE, a recent trial which compared TACE followed by external beam radiation therapy (EBRT) to sorafenib, in patients with macrovascular invasion, demonstrated a significantly longer time to progression (TTP) (31 weeks) and OS (55 weeks) in the TACE plus EBRT cohort [35]. However, while these trials demonstrate promising responses when compared to historical studies, they certainly leave significant room for improvement.

### 6.1. Combination of Immunotherapy and Locoregional Therapies in HCC

The general concept of helping to “prime” the immune system to induce increased clinical response to checkpoint inhibitors has been of interest for several years now [36]. While these concepts have been well studied and even put into practice in the setting of EBRT, they are less well known when it comes to locoregional therapies [37,38]. However, with the introduction of checkpoint inhibitor treatments into the treatment algorithm for HCC patients, greater interest has been created and data have begun to be produced [39,40]. Furthermore, given the excitement of these potential synergisms and the limited options for patients, some multidisciplinary teams have begun offering these options to well selected patients.

While an in-depth analysis of how checkpoint inhibitors work is outside the scope of this review, a brief synopsis will be provided. Then, a more detailed review of the available, perhaps less well known, data on the immunomodulatory properties of locoregional therapies with and without the addition of immune checkpoint blockade will be summarized. Finally, a brief discussion of the combination of locoregional therapies and other immunomodulatory methods will be provided.

Prevention of self-harm through the avoidance of the immune system attacking “normal tissue” is a fundamental aspect of the immune system. To this end, the immune system has developed checkpoints that serve to suppress the immune response, for instance, when self-antigens are presented [41,42]. There are numerous checkpoints that occur to keep the immune system from attacking self, and this process is commonly referred to as peripheral tolerance [43,44]. Unfortunately, tumor cells can utilize these checkpoints as well to avoid detection by the immune system [45,46]. It is important to remember that the immune system is constantly surveying and eliminating cells that become aberrant. However, in settings of chronic inflammation and early cancer development, immune exhaustion is typically present. Immune exhaustion leads too decreased effector cytokine production and cytolytic activity of CD 8+ T cells. It also leads to an overexpression of inhibitory receptors, such as programmed death 1 (PD1). The state of immune exhaustion is typically characterized by an overabundance of regulatory T cells.

Over the last several decades, two checkpoints, the cytotoxic T lymphocyte-associated antigen-4 (CTLA-4) and PD1 pathways, have been highlighted for their importance in cancer immunology with the development of checkpoint inhibitors as effective treatments for multiple cancers, through the blockade of these pathways. The CTLA-4 pathway functions to stop potentially autoreactive T cells during the initial stages, namely, the naïve T cell activation, which typically takes place in the lymph nodes [44,47]. On the other hand, the PD1 pathway functions to regulate previously activated T cells during later stages of the immune response, which in turn typically takes place in the periphery [42,44]. To date, the checkpoint inhibitors available on the market have served to block one of these pathways and thus allow the immune system to detect and attack the cancer. However, as discussed above, while representing a monumental step forward, the response rates are not perfect and opportunities for improvement are of interest.

### 6.2. Locoregional Therapies and the Abscopal Effect

**Figure 1:**
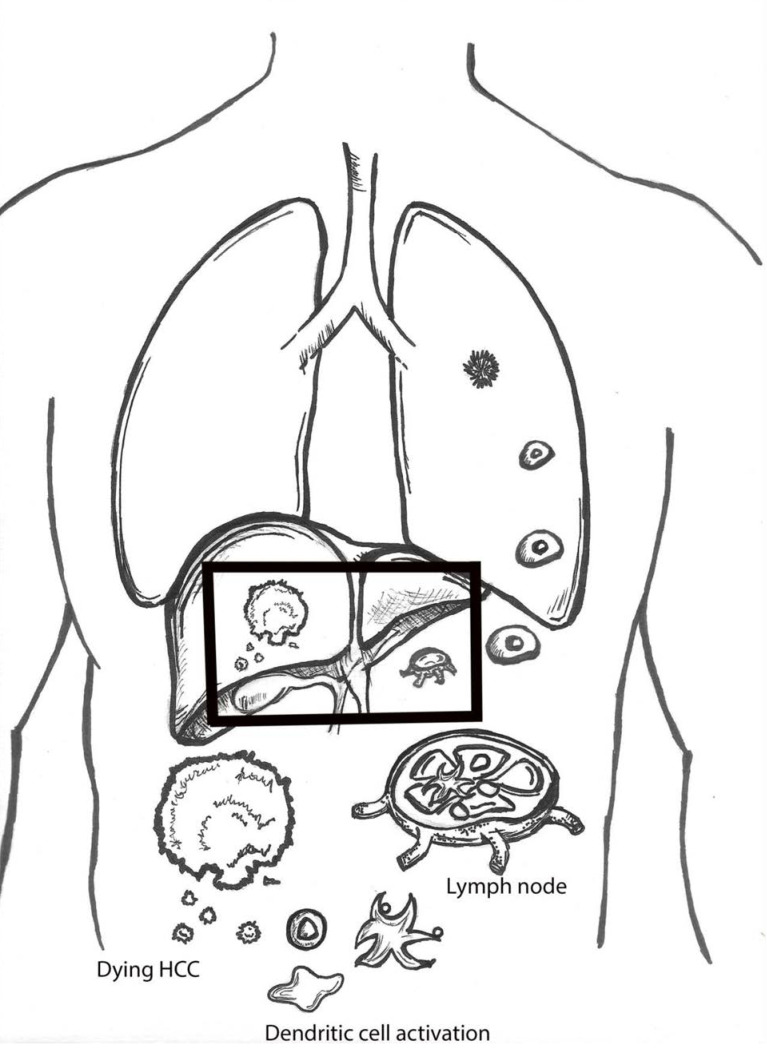
Abscopal effect demonstrating the activation of the immune system after hepatocellular carcinoma (HCC) cell death.

**Figure 2:**
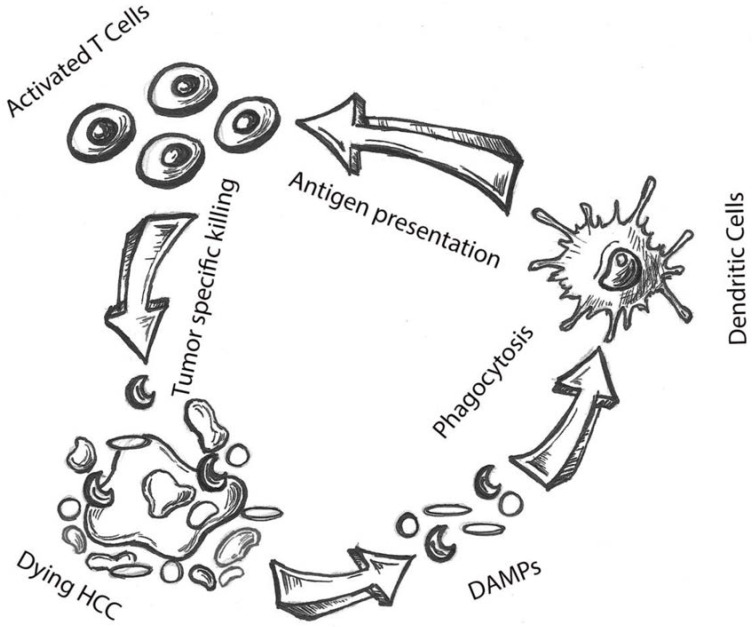
Cycle of dying hepatocellular carcinoma (HCC) cells resulting in the release of damage-associated molecular patterns (DAMPS), followed by DAMPS leading to increased antigen presentation within dendritic cells. Antigen presentation within dendritic cells in turn leads to activation of T cells that results in more tumor-specific cell killing.

The ability to induce the immune system to attack off-target lesions after treatment of a lesion is commonly referred to as the abscopal effect (Figure 1). The abscopal effect represents the holy grail for locoregional therapies as it allows them to exert systemic effects despite only delivering local treatments. Abscopal effects are a manifestation of the immune activation sometimes observed after tumor cell death (Figure 2). While abscopal effects have been observed after locoregional therapy in HCC patients, they are by no means common place. Therefore, the interest of adding other treatment strategies, such as systemic therapies, is crucial to induce these effects more frequently.

### 6.3. Ablation

Ablation is a broad term that includes specific techniques such as RFA, microwave ablation (MWA), irreversible electroporation (IRE), and cryoablation among others. However, perhaps of these the most data exist in understanding the immunomodulatory abilities of RFA and cryoablation [48].

To understand how ablation leads to immunologic activation, it is beneficial to understand how it induces cell death at a molecular level. For instance, apoptosis in MWA and RFA is a result of heat. Heat-induced apoptosis results from activation of intercellular signaling, eventually resulting in DNA fragmentation. It is known that sphingomyelinase activation occurs, ceramide concentration changes, and finally stress-activated protein kinases/c-Jun N-terminal kinases’ (SAPK/JNK) activation takes place. This signaling leads to cysteine-directed asparagine proteases (caspases) activation [49]. The caspases family are in turn known to play important roles in which form of apoptosis (necroptosis, phyroptosis, etc.) is ultimately undergone by the cell, which has immunologic implications [50]. On the other hand, while cryoablation is often thought of as a relatively simplistic means of creating cell death with disruption of the blood supply and cell membrane, in fact cryoablation induces cell death by a number of different means [51]. One is persistent hypothermia that leads to uncoupling of most metabolic pathways, depletion of adenosine triphosphate (ATP), ionic imbalances, cellular acidosis, and free radical generation. Not only is the cellular membrane disrupted but also cellular organelles as well experience a change in fluidity and disassembly of cytoskeletal structures [52]. Ultimately, necrosis with structural damage, stress-induced apoptosis, and tumor hypoxia results in cell death by a number of causes. Cellular breakdown leads to proinflammatory cytokines, DNA, RNA, and HSP being released for an extended period of time. These complex mechanisms lay the foundation for the immunologic changes observed following ablation and are discussed below.

RFA has been shown to activate the acquired immune system through the release of intracellular components following treatment [53,54]. Of the array of neoantigens released, many of which have immunologic effects, HSP may be of particular interest. While helping to prevent apoptosis when intracellular but once they have entered the extracellular environment, HSPs have been shown to activate an acquired immune response [55,56,57]. HSPs can serve to chaperone antigens for presentation to dendritic cells (DC) and also facilitate activation of DCs [56,57]. Of the HSPs, HSP 70 has been shown to be elevated in serum of patients following RFA [58]. It is also possible that RFA reduces immune tolerance of tumor cells as it has been shown to reduce levels of regulatory T cells [59]. Furthermore, RFA has been shown to increase intratumoral T cells, tumor-specific antibodies, and CD4+ and CD8+ cells following ablation [60,61].

The immunomodulatory effects of cryoablation have been of interest for several decades now. Cryoablation has been shown to lead to the production of anti-tumor antibodies [62,63]. Furthermore, lymphocytes harvested after cryoablation have been shown to be tumor specific, and animal models have indicated that cryoablation provides some resistance to cancer [64,65]. In a murine model, cryoablation was shown to induce a tumor-specific T cell response in tumor-draining lymph nodes as well as an increased systemic natural killer (NK) cell activity, which correlated with the rejection of tumors on rechallenge [65]. Cryoablation has also been shown to induce a greater post-ablative immune response, as compared to RFA and MWA, in terms of immunologically active cytokines and DC response [54,66,67].

While less data is available, discussion of the potential of IRE and histotripsy is likely warranted. IRE induces death through high voltage short electrical pulses [68]. It has been shown to release significant intracellular proteins and lead to substantial T cell activation [68,69]. Furthermore, IRE has been shown to lead to increased CD3+ T cells, CD8+ T cells, DC, and macrophages while decreasing regulatory T cells in the microenvironment of treated tumors [69,70,71,72]. While some of the technical challenges of an IRE procedure have limited widespread adoption, improvements in the technology may mitigate some of these concerns. Histotripsy induces mechanical disruption of the cellular membrane, and while no commercially available devices are available, some promising early data have been produced, suggesting immune manipulation may be possible [73].

The previously discussed data have led to interest in developing trials that attempt to leverage antitumor effects of both ablation and checkpoint inhibitors [74,75,76]. Table 1 reports on selected studies that evaluate the combination of locoregional therapies and immunotherapies. As proof of concept, a study investigated 32 patients with advanced HCC receiving 6 doses of tremelimumab, anti-CTLA-4 antibody, followed by infusion every 3 months. These patients underwent RFA or chemical ablation on day 36, demonstrating a relatively modest median OS of 12.3 months and PFS of 57.1% and 33.1% at 6 and 12 months, respectively [77]. An increase in CD8+ T cells at the 6-week tumor biopsy was observed in responders only. Furthermore, searching ablation “and” PD1 on clinicaltrials.gov found 52 studies as a result, indicating the interest for further data in this area. However, it should be noted that while the activation of the immune system has been the focus of the above section, some data have shown that at times ablation can induce a more tumor-tolerant immune environment. This emphasizes the need for further research and understanding of the mechanistic actions of the treatments.

### 6.4. Transarterial Chemoembolization (TACE)

Again, molecular signaling is important to understand in regard to post-TACE immunogenic response. TACE induces cell death by ischemia and by the introduction of large amounts of chemotherapy, typically Doxorubicin. Doxorubicin has been shown to promote cellular death through both caspases-dependent and independent mechanisms and is furthermore not dependent on the FAS/extrinsic cell death signaling pathway [84,85]. Ischemia, related to the disruption of blood flow after particle introduction, leads to depletion of ATP. This in turn leads to impairment of ionic pumps, cell swelling, clearing of cytosol, dilation of the endoplasmic reticulum and golgi apparatus, mitochondrial condensation, chromatin clumping, and cytoplasmic bleb formation [86]. Both forms of cellular death become important in understanding the immune reaction, which is observed following TACE.

While relatively fewer studies have investigated the immunomodulatory effects of TACE, several publications have been produced. TACE has been shown to induce production of the pro-inflammatory cytokine interleukin (IL) 6, but also of suppressive cytokines [87]. TACE has also been shown to increase the CD4+/CD8+ T cell ratio and the frequency of tumor-specific CD4+ T cells [86,87]. Finally, TACE has been shown to increase the PD1 expression in peripheral mononuclear cells, while also decreasing the number of peripheral regulatory T cells [88,89,90].

While the true ability of TACE to modulate the immune system both systemically and locally in either a positive or negative way remains largely unknown, significant interest in combining TACE with or without EBRT with checkpoint inhibitors has been shown [78,79,80]. In a recent prospective trial, Chiang et al. showed promising results of combining EBRT, TACE, and avelumab (anti PD-L1) for locally advanced HCC. In a cohort of 33 patients where 64% had macroscopic vein invasion, they were able to convert 12% to curative intent treatment and achieve a complete radiologic response in 42% [78]. In another interesting study, Yang et al. found that the addition of TACE to regorafenib and checkpoint inhibitor treatment achieved a longer PFS and OS as compared to regorafenib and a checkpoint inhibitor alone [79]. However, it should be noted that both the reference studies above and the majority of similar studies are coming from Asia. Asia has a significantly different HCC patient population to other areas of the world, and it is notable that TARE is not commercially available in the majority of these areas.

### 6.5. Transarterial Radioembolization

Molecular aspects of cell death following radiation are perhaps the most studied of the entities described here. Ionizing radiation leads to DNA damage, both from direct action as well as from the formation of free oxygen radicals [91]. When the single- and double-strand breaks become too great for the ataxia telangiectasia mutated (ATM) and other DNA repair mechanisms to repair, cell death is induced. One important aspect to remember is that some cells may avoid cell death and instead enter senescence where the cell terminates division processes through p53/p21 and p16/RB1 signaling [92]. These cells not only remain viable but do not contribute to the induction of an immune response. Those cells undergoing cell death do so by either necrosis or programmed cell death depending on the extent of damage. While all cell death has some component of immunogenicity, necrosis is typically thought of as more immunogenic and thus more desirable to induce positive immune responses discussed below.

TARE has invoked strong optimism early in the development of checkpoint inhibitors, given the potential of synergism with them. This enthusiasm has in part been based on the principle established in preclinical models with EBRT, which has demonstrated that increasing the radiation dose to the tumor also increases the likelihood of inducing positive immune responses such as the abscopal effect [93,94]. Significantly higher doses can be delivered to the tumor with TARE as compared to EBRT in clinical settings [95]. However, at the same time, it should be noted that the radiation given in TARE and EBRT differs, and it is well known that inducement of an abscopal effect is easier to achieve in murine models as compared to humans. Nonetheless, enthusiasms remains high, and we review the available data below. Because of the robust data confirming the ability of radiation to positively modulate the immune system in preclinical models, we focus on the human data herein.

In perhaps the most robust data of human immunomodulation by TARE, Chew et al. evaluated the tumor tissues, including tumor infiltrating lymphocytes, and peripheral blood monocytes using time-of-flight mass cytometry and next-generation sequencing before and at 1, 3, and 6 months following TARE in 44 HCC patients [96]. In these patients who underwent surgical resection after downstaging with TARE, the study found that treated tumors were enriched with CD56+ NK cells, CD8+CD56+ NK T cells, CD8+ T cells, and CD4+ T cells. Furthermore, the treated tumors had an enrichment in the number of GB+CD8+ T cells and Tim-3+CD8+ T cells, which were infiltrating the tumor as compared to controls. Treated tumors also had a larger percentage of CXCR3-expressing CD4+CD45RO+ T cells and a smaller percentage of immunosuppressive Foxp3+CD152+CD4+ regulatory T cells as compared to controls. The number of antigen-presenting cells was similarly increased, and the overall findings seem to suggest that TARE may be able to facilitate the creation of more immune-active tumor microenvironments. These findings were fairly similar to evaluations of EBRT on lung cancers in humans, suggesting that there is further reason to believe that the same positive signals observed in EBRT preclinical models may be applicable to TARE as well [36,97]. Chew et al. also demonstrated an increase in PD1-expressing CD8+ and CD4+ T cells in patients with sustained response 3 months following TARE as compared to non-responders [96]. This finding was similar to a study by Rivoltini et al. who demonstrated significant increase in CD3+PD-1+ lymphocytes and PD-L1+ monocyte populations 1 month after TARE in 49 HCC patients [98]. They did decrease in these populations at 3 and 6 months, leading the authors to suggest that perhaps the introduction of checkpoint inhibitors may prolong the favorable immune environment. Finally, a study of 23 patients receiving TARE for metastatic breast cancer showed that increased frequency of baseline PD-1 expression by CD4+ TILs in the tumor microenvironment was associated with clinical response [99]. While this study is in a different cancer population than HCC, it does provide some further reason to be optimistic about the underlying scientific principle. Furthermore, TARE has been shown by several authors to positively impact the cytokine profile observed in HCC patients after treatment [100,101,102].

These results have led to enthusiasm for combining checkpoint inhibitors and TARE, in the setting of HCC. In some of the first-published data, which primarily focused on safety, a retrospective study of 26 patients who were administered a checkpoint inhibitor within 90 days of TARE treatment demonstrated no grade 3/4 hepatobiliary or immunotherapy-related toxicities [81]. The study also demonstrated an OS of 16.5 months and a TTP of 5.7 months as measured by the first TARE. In a prospective trial, 40 patients with HCC were treated with TARE, followed by nivolumab 21 days after TARE and every 2 weeks after that [82]. Two patients experienced grade 3/4 treatment related adverse events that were all laboratory in nature, while 5 had serious adverse events. The patients showed an objective radiologic response rate (ORR) of 30.6%, leading the authors to suggest further study of this combination. Finally, a search of “TARE OR SIRT OR yttrium 90 AND PD1” on clinicaltrials.gov revealed 225 studies, indicating the enthusiasm for this line of study.

## 7. Other Immune-Activating Therapies

The efficacy of checkpoint inhibitors in HCC may be limited due to multiple immune evasion mechanisms, including the generalized immunosuppressive environment of the cirrhotic liver as well as T cell exhaustion. In the future, direct injection of alternative immune-stimulating agents into HCCs may be used to augment locoregional or systemic approaches. Toll-like receptor 9 (TLR9) agonists (CpG) have been developed to stimulate the innate arm of the immune system [103]. Recently, phase I dose escalation trials involving the intratumoral injection of CpG into refractory visceral solid tumors alone or in combination with ipilimumab demonstrated promising results [104,105]. Furthermore, a combination of intratumoral injection of TLR9 agonist and OX40 agonist, a T cell co-stimulating molecule, showed an antitumor immune response in a mouse model of HCC [106]. In parallel, there has been much interest in the development of oncolytic viruses (OVs) in preclinical and early clinical studies [107]. OVs can drive oncolysis directly but can also be engineered to express genes that may upregulate the immune response, such as granulocyte macrophage colony stimulating factor (GM-CSF). In a Phase I trial, three HCC patients underwent a series of direct intratumoral injections of JX-594, a targeted and GM-CSF-expressing oncolytic poxvirus, followed by systemic Sorafenib [108]. Three to six month follow-up imaging showed decreased tumor perfusion and increased necrosis [108]. Several groups have also started to evaluate the potential of these mechanisms when combined with ablation. While early in development, these strategies may provide opportunities to HCC patients in the future.

## 8. Future Directions

The rationale for the combination of locoregional and immunotherapies is well founded with early clinical data seeming to support the signals observed in the preclinical space. HCC has the potential to be an area where these synergistic treatment protocols lead to significant improvements in patient outcomes. However, further data is desperately needed to determine a number of different critical aspects. For instance, the sequence of intervention that would be the most advantageous remains debatable. Furthermore, greater understanding of the immunomodulatory effects of the locoregional therapies would be of benefit, particularly in the setting of TARE where factors such as tumor and normal tissue dose may play important roles in outcomes. Finally, patient selection remains an important clinical consideration. The emphasis for the importance of more data to guide treatment strategies is underlined by the fact that many multidisciplinary teams have already initiated combination strategies in some form.

## 9. Conclusions

While there are certain areas that remain clearly the domain of locoregional or systemic therapies, the area of greatest research activity and interest in the current landscape is that of locally advanced HCC where both modalities may come into play. While it is possible to take a position to support one or another in these instances, it is very likely that a sequential or combination treatment will be proven to be most effective. Furthermore, patients with confirmed extra-hepatic disease present another potential area for combination therapy; however, it remains unstudied. The need for further clinical data in this area is clear, and the authors hope more studies will be forthcoming soon.

## Figures and Tables

**Table 1:** Selected studies evaluating the combination of locoregional therapies and immunotherapies.

Study	Description
Ablation	
Duffy et al. [77]	Single-arm/institution prospective study of tremelimumab with subtotal RFA performed on day 36 in HCC patients. Noted increase in intratumoral CD8^+^ T cells and an acceptable safety profile.
Qiao et al. [76]	Prospective study of patients who underwent TACE + MWA. Patients were offered ICI, and 15 patients accepted ICI, and the other 21 were utilized as a control group. Interim analysis demonstrated a prolongation of RFS in those who had ICI in addition to TACE + MWA.
TACE	
Chang et al. [78]	Prospective single-arm study of TACE, followed by EBRT, and followed by avelumab in 33 patients with locally advanced HCC. Demonstrated favorable oncologic outcomes and complication profiles.
Yang et al. [79]	Retrospective study of 52 HCC patients treated with second-line therapy, comparing regorafenib plus ICI plus or minus TACE. Demonstrated improved OS in patients who had TACE in addition to regorafenib and ICI.
Yuan et al. [80]	Retrospective multicenter study of HCC patients with portal vein tumor thrombus who underwent R0 resection and compared to those that received post-surgical TACE and those that received post-surgical TACE and ICI. Study found TACE plus ICI prolonged OS and RFS as compared to those who received only TACE in the post-surgical time period.
TARE	
Zhan et al. [81]	Retrospective single center study of 26 patients who underwent TARE followed by ICI. The study focused on safety and the study suggested safety of the combination.
Tai et al. [82]	Prospective single-arm study evaluating TARE followed by nivolumab 21 days after and every 2 weeks thereafter. The objective response rate was positive and the safety profile was acceptable.
de la Torre-Alavez [83]	Single-arm prospective multicenter study in HCC patients treated with TARE followed by nivolumab 3 weeks later. Study demonstrated promising oncologic outcomes and safety profile.

RFA = Radiofrequency ablation, HCC = hepatocellular carcinoma, RFA = radiofrequency ablation, MWA = microwave ablation, TACE = transarterial chemoembolization, EBRT = external beam radiation therapy, ICI = immune checkpoint inhibitor, OS = overall survival, RFS = recurrence-free survival, TARE = transarterial radioembolization.

## Data Availability

Not applicable.
